# Excess of transmission of the G allele of the -1438A/G polymorphism of the 5-HT_2A _receptor gene in patients with schizophrenia responsive to antipsychotics

**DOI:** 10.1186/1471-244X-8-40

**Published:** 2008-05-30

**Authors:** Dalila Benmessaoud, Nora Hamdani, Claudette Boni, Nicolas Ramoz, Michel Hamon, Farid Kacha, Philip Gorwood

**Affiliations:** 1 Etablissement Hospitalier Spécialisé Psychiatrique M. Boucebci. Cheraga, Alger, Algeria; 2 INSERM U675, Faculty of Medicine Bichat (IFR02), 16 rue Henri Huchard, 75018 Paris, France; 3 AP-HP, Hôpital Louis Mourier (Paris VII), service de psychiatrie, 178 rue des Renouillers, 92700 Colombes, France; 4 UMR 677 INSERM/UMPC, Neuropsychopharmacologie, IFR70 des Neurosciences, CHU Pitié-Salpêtrière, 75013 Paris, France

## Abstract

**Background:**

The -1438A/G polymorphism of the 5-HT_2A _gene has been found to be associated with clinical response to clozapine and other second generation antipsychotics. Testing the impact of this marker on response to first generation antipsychotics (which have a lower affinity for the 5-HT_2A _receptor) provides the opportunity to help disentangling the two different roles that this polymorphism might have. A psychopharmacogenetic role should be detected only for antipsychotics with high affinity to the 5-HT_2A _receptor (therefore to second generation antipsychotics). An alternative role would imply tagging a subgroup of patients responsive to any antipsychotic, whatever their affinity, meaning that the association is more depending on non pharmacological charaterictics, such as clinical specificities.

**Methods:**

A family-based sample of 100 Algerian patients with schizophrenia (according to DSM-IV criteria) and their 200 biological parents was recruited, in order to avoid stratification biases. Patients were all treated, or have been treated, by conventional antipsychotics (mainly haloperidol) for at least four weeks, at appropriate dosage. May and Dencker scale was used to distinguish responders and non responders.

**Results:**

No allele of the -1438A/G polymorphism of the 5-HT_2A _gene was transmitted in excess (50 transmitted for 38 untransmitted) in the whole sample of patients with schizophrenia (p = .90). In contrast, a significant excess of transmission of the G allele was observed (p = .02) in the subgroup of patients with good treatment response (17 transmitted for 6 untransmitted).

**Conclusion:**

Using a TDT approach, we showed that the G allele of the -1438A/G polymorphism of the gene coding for the 5-HT_2A _receptor was associated to schizophrenia with good response to conventional antipsychotics, although this conclusion is based on 88 informative patients only. Because previous data showed the same result with atypical antipsychotics, it can be concluded that the G allele tags a subgroup of schizophrenic patients with greater chance of improvement with antipsychotics of either type.

## Background

There are large inter-individual differences in the clinical efficacy of antipsychotics in schizophrenia. Age at onset of schizophrenia, severity of the disorder, presence of extra-pyramidal symptoms and tardive dyskinesia may be involved in such variability [1-4]. Psychopharmacogenetics may give valuable clues to potentially help the clinician to prescribe the most appropriate treatment for each patient, notably by allowing an estimate of individual risk of antipsychotic refractoriness or severe side effects [5]. Based on the pharmacological profile of second generation antipsychotics, one of the most frequently studied genes in this domain has been the gene coding for the 5-HT_2A _receptor. Indeed, as compared to conventional antipsychotics, second generation (or atypical) antipsychotic drugs have higher affinity for this receptor than for the dopamine D2 receptors [6]. The 5-HT_2A _gene is located at chromosome region 13q14.2 in a locus that has been linked with schizophrenia [7], but with conflicting results regarding association studies [8]. Two common single nucleotide polymorphisms (in almost complete linkage disequilibrium) of this gene have already been tested in schizophrenia, a silent variation in exon 1 (T102C) and an upstream polymorphism in the promoter (-1438A/G). Both T102C and -1438A/G SNPs may affect 5-HT_2A _receptor expression, in a tissue-specific manner [9].

The T102C polymorphism of the 5-HT_2A _gene has been associated with good clinical response to clozapine in one study [10], but not in five others [11-16]. According to Arranz et al. [17,18], the second polymorphism (-1438A/G) would also be associated with the quality of response to clozapine. These authors reported that 373 patients (extracted from six different studies), that responded well to clozapine had a moderate but significant excess of AA genotype (OR = 1.36) compared to 360 non-responders [18]. In contrast, a later study on a large Asian population of schizophrenic patients, treated with risperidone, another second generation antipsychotic, showed a better outcome for patients with the GG genotype [19]. The exact role of the 5-HT_2A _gene in the response to treatment with second generation antipsychotics is therefore difficult to assess on the basis of such heterogeneous data.

Case control studies are exposed to population stratification bias, i.e. allele variability may be explained by differences in ethnic background of the compared populations, rather than the quality of a trait such as treatment response. Although more difficult to perform, family based association studies offer an interesting approach, as they are protected from such bias. However, to date, such studies have only been rarely undertaken for psychopharmacogenetic purpose.

On the other hand the exact role of a candidate gene in a complex disorder is always difficult to determine. For example, we showed that both positive and negative symptoms of schizophrenia have to be taken into account when assessing the possible role of the 5-HT_2A _gene in treatment response. In our sample, the -1438 A allele of the 5-HT_2A _gene was found to be associated with the severity of negative symptoms, which by itself was sufficient to predict refractoriness [20]. Accordingly, careful consideration to the clinical specificities of the sample may help disentangling the exact role of a candidate gene regarding vulnerability/resistance to a disease or its response to treatment.

In this context, it is surprising that the role of the 5-HT_2A _gene in the response to first generation antipsychotics has so rarely been investigated. Indeed, if the -1438 A allele is tagging a subgroup of patients with lower chance of treatment response [19], then it is expected that this allele would have a similar role in case of treatment with all antipsychotics, independently of their high or low affinity for the 5-HT_2A _receptor. In contrast, if the -1438 A allele is more directly involved in the mechanism of action of second generation antipsychotics, then we should observe no role of this gene in the response to first generation antipsychotics with low affinity for the 5-HT_2A _receptor.

In the present family-based association study, we directly addressed this question by quantifying -1438A/G polymorphism in a sample of schizophrenic patients treated with conventional antipsychotics (mainly haloperidol) and their parents. We used a Transmission Disequilibrium Test (TDT) considering the observed transmission of the marker from heterozygous parents to the affected offspring compared to expected transmission (50%). This approach increases the power to uncover a linkage [21]. Our hypothesis was that the -1438A/G polymorphism was associated with treatment response also in patients treated with first generation antipsychotics.

## Methods

### Clinical sample

The sample consisted of 101 Algerian patients with schizophrenia and their 202 biological parents. All subjects were of Algerian origin and had a diagnosis of schizophrenia according to DSM-IV criteria.

The inclusion criteria required that patients were all treated, or had been treated, by conventional antipsychotics (mainly haloperidol) during at least four weeks at an appropriate dosage. All the recruited subjects gave informed consent for their participation in the study, which was approved by the national ethical committee of Algeria. As first generation antipsychotics were the first line treatment for schizophrenia in Algeria during the recruitment of this sample (year 2005), only one patient (1%) was excluded because of treatment with second generation antipsychotics. The final sample is therefore made of 100 patients exactly and their 200 parents.

All informations were obtained from a face-to-face systematic interview by a trained psychiatrist (DB), enriched by hospital and ambulatory case notes. Treatment response was systematically assessed, retrospectively and at the moment of the clinical interview, using the six point May and Dencker scale [22]. Levels 1 and 2 might indicate "remission", with no need for a formal rehabilitation program. Levels 3 and 4 signify responsiveness to a learning-based rehabilitation program. Level 5 suggests the requirement of continuous, individually oriented, strategies, including intensive trials of antipsychotics. Level 6 is chosen for patients needing long-term hospitalization [22].

Scores above 3 were considered as reflecting refractoriness, and scores below 4, responsiveness, to allow comparison with our previous study [20]. Clinical information was collected using the D.I.G.S., a semi-structured interview [23] commonly used in psychiatric genetics for the assessment of substance abuse/dependence and lifetime clinical characteristics of schizophrenia. Negative and positive symptoms of schizophrenia were evaluated using the SANS and SAPS scales [24], while current extrapyramidal symptoms were assessed with the Simpson & Angus scale [25].

### Statistics

The TDT and the family-based association test (FBAT) were applied to assess the association of the -1438A/G polymorphism of the 5-HT_2A _gene with schizophrenia and response to first generation antipsychotic [26]. Determination of the required sample size was based on both the frequency of the G allele (42% in our previous sample; [20]) and an estimated genotype relative risk (gamma) of 1.36 (the one calculated in the meta-analysis of case-control studies [18]). With such values, 110 trios are needed [27] in order to detect any significant excess (p < 0.05) of transmission of one allele. For quantitative and binary variables, potentially transmitted with the -1438A/G polymorphism, the PBAT test was used [28].

### 5-HT_2A _receptor gene polymorphism

Blood was collected after the interview and genomic DNA was isolated from leucocytes using standard salting out procedures [29]. Five independent SNPs (not located on the same chromosome) were tested to check paternity, without being able to exclude any.

The -1438A/G polymorphism located in the promoter of the 5-HT_2A _receptor gene was determined using a polymerase chain reaction as previously described [30].

## Results and discussion

One hundred complete trios with schizophrenic probands (81 males and 19 females) participated in the study. Thirty eight patients were considered as good responders while 62 were not. The non responsive group was predominant, probably because of the highly demanding criteria of the May and Dencker scale, and exhibited significantly higher scores for positive (p < 0.001), negative (p < 0.001) and extra-pyramidal symptoms (p < 0.001) (Table 1).

**Table 1:** Demographic and clinical charateristics of the 100 patients with schizophrenia according to good versus poor response to antipsychotics (May and Denker classification).

Clinical variables	Poor responders (N = 65)	Good responders (N = 38)	p
Men (%)	77	87	0.445
Age at interview (mean ± sd)*	32.83 ± 7.89	31.33 ± 7.21	0.368
Age at onset (mean ± sd)*	21.66 ± 5.02	21.03 ± 3.45	0.464
Illness duration (mean ± sd)	11.17 ± 8.16	10.29 ± 6.78	0.554
SAPS scores (mean ± sd)	5.14 ± 3.75	1.35 ± 2.84	< 0.001
SANS scores (mean ± sd)	12.11 ± 4.62	9.02 ± 4.29	< 0.001
Extra-pyramidal scores (mean ± sd)	2.86 ± 3.63	0.92 ± 1.55	< 0.001

* years

**Figure 1:**
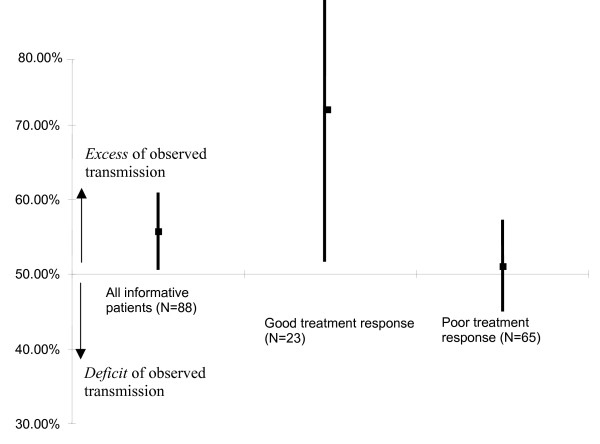
Percentage of observed (versus expected) transmission of the G allele (and their 95% CI) of the 5-HT_2A _gene in 100 patients with schizophrenia (with 88 informative ones having at least one heterozygous parent), and in two subgroups according to clinical response to first generation antipsychotics.

The genotype distribution of the trios was in accordance with Hardy-Weinberg equilibrium, as well as that of each of the two subgroups of patients with good versus poor response to treatment. No allele of the -1438A/G polymorphism was transmitted in excess in the whole sample of patients with schizophrenia (Mc Nemar χ^2 ^= 1.63, p = 0.20). However, an excess of transmission of the G allele was observed in the responsive group (Mc Nemar χ^2 ^= 5. 26, p = 0.02) but not in the non-responsive group (Mc Nemar χ^2 ^= 0.01, p = 0.90) (Table 2, Figure 1), the number of informative transmissions being limited (23 good responders versus 65 poor responders).

**Table 2:** Transmission disequilibrium test (TDT) for the G allele of the -1438A/G polymorphism of the 5-HT_2A _gene in a sample of 100 trios with schizophrenia according to good versus poor response to antipsychotics (May and Denker classification).

Sample	G allele		χ^2^	p
			
	Transmitted	Untransmitted		
				
All patients	50	38	1.63	0.20
Good treatment response	17	6	5.26	0.02
Poor treatment response	33	32	0.01	0.90

Major clinical variables did not apparently interfere in the association between the G allele and the group of patients responsive to treatment, as we found no association, according to the PBAT test, between the G allele and duration of illness (p = 0.25), negative (p = 0.16), positive (p = 0.41) and extrapyramidal (p = 0.41) symptoms, nor with the age at onset (p = 0.96). Therefore, it can be concluded that the TDT strategy allowed us to show that the G allele off the -1438A/G polymorphism was transmitted in excess among schizophrenic patients responsive to first generation antipsychotics (haloperidol) in our sample. Interestingly, an excess in G allele transmission was also noted in our previous study based on a case-control approach in schizophrenic patients treated by second generation antipsychotics [20]. Because this association was also detected for amisulpride (which has a very low affinity for 5-HT_2A _receptor) in the latter study, and the risk of stratification bias was eliminated in the present study, it can be reasonably inferred that the 5-HT_2A _gene could be involved in clinical or other traits tagging specific subgroups of patients, with higher chance of improvement by treatment with various antipsychotics, whatever their affinity for 5-HT_2A _receptors. As some first generation antipsychotics may exert some moderate but meaningful antagonism at this receptor, and as the effects of antipsychotics may not depend on receptor antagonism, but rather on intracellular mechanisms, alternative explanations can be proposed.

As positive and negative symptoms as well as extra-pyramidal symptoms were greater among poorly responsive patients, these clinical features might have acted as intermediate factors between the 5-HT_2A _gene polymorphism and response to treatment. In fact, no link was found between these factors and the G allele of the -1438A/G polymorphism of the 5-HT_2A _gene using specific measures. However, it has to be emphasized that the detection of such a link in this sample of trios is less obvious than in our previous case-control study (although both samples included roughly the same number of probands[20]: N = 106; present study: N = 100) which allowed the inclusion of all patients (N = 64 responsive patients). With the TDT, only patients from at least one heterozygous parent and who exhibited good response to treatment can be used (N = 23 potential transmissions). Furthermore, the required sample size was a bit over the one we had in this study (i.e. we reached only 91% of the targeted recruitment). Therefore, the chance to detect such a link is greatly reduced, even when using quantitative trait loci (QTL) with the PBAT software. Difference of statistical power was already raised in one positive case-control study but it was negative in the familial association study concerning the T102C polymorphism of the 5-HT_2A _gene in schizophrenia [31]. Furthermore, the TDT approach is based on the assumption that both participating parents are the biological ones, which might be wrong for a significant part of the fathers. Even if we probably decreased this risk by testing 5 independent SNPs, more informative markers (i.e. VNTR) on a larger set of genes (i.e. more than 5) would have been more appropriate to detect exclusion of parternity.

The -1438A/G polymorphism was found to be associated with first generation antipsychotic efficacy in our sample. Efficacy in this sample was relying on May and Dencker scale, which is based on past treatments. Retrospective analysis may have different advantages, as allowing the assessment of longer periods, the comparison of the efficacy of different treatments in a single patient, and being protected from the problem of follow-up (with the associated attrition rate). On the other hand, such assessment is more exposed to different confounding parameters such as an uncontrolled length of exposition and dosage to different treatments, and a lower validity of assessment. These limits should be taken into account. Comparison with available data in the literature cannot really be made because the largest part of psychopharmacogenetics studies on antipsychotics in schizophrenia concern second generation antipsychotics. Furthermore, reported studies mainly relied on the T102C polymorphism. Nevertheless, it is interesting to note that the meta-analyses of antipsychotic response by Arranz et al. [17,18], and of schizophrenia *per se *by Abdolmaleki et al. [8], are in favor of a significant role of the C allele of the T102C polymorphism, which mainly corresponds to the G allele of the -1438A/G polymorphism, as these two markers are in nearly complete linkage disequilibrium. The conclusion would then be that the allele which was associated with good response to antipsychotics and the risk of schizophrenia was in fact less frequent in our sample of patients with good response. Three points have to be raised in front of such discrepant results.

Firstly, in meta-analyses, large inter-studies variability of the G allele frequency was detected, with a tendency in favour of an association with the other allele (equivalent to the A allele of the -1438A/G polymorphism) in samples of non-Caucasian subjects. However, a large prospective study in an Asian sample [19] showed that the G allele was associated with good response, in accordance with our previous study [20] and the data reported herein. Such discrepancy in the results can be ascribed to the variability of the G allele frequency in different ethnic backgrounds, and tends to favor intra-familial, rather than case-control, association studies. In any case, because of the rather modest number of informative transmissions (50 allelic transmissions among the 200 available for the 100 trios, and 17 allelic transmissions of the 76 available in the responsive group), our results should be interpreted with caution [27], the risk of chance finding being high. Using more tagging single nucleotide polymorphisms (tagSNPs), including the T102C polymorphism, would improve the statistical power of the analyses (because of the higher rate of heterozygous parents) and would help to pinpoint which haplotype might be involved in treatment response for this gene. Furthermore, the probability that different SNPs from many other genes are involved in treatment response is high, in accordance with the relatively small attributable risk usually detected in psychopharmacogenetic studies.

Secondly, as recalled above, the majority of pharmacogenetic studies were performed on second generation antipsychotics, and mainly on clozapine, in accordance with the need to identify patients that can benefit of such effective but potentially life-threatening treatment. This implies that mostly severe and resistant patients have been included in these studies, in contrast with our sample and that of the Lane et al. [19] study which comprised both good responders and poor responders to antipsychotics. Similarities in the recruitment of the latter two samples probably explain why the data of Lane et al. [19] and ours are convergent.

Thirdly, the impact of the G allele of the -1438A/G polymorphism of the 5-HT_2A _gene is not yet clear. Many studies propose that this polymorphism might allow prediction of resistance to treatment [18], while others claim that the G allele could be an independent risk factor for schizophrenia [8]. Our data suggest that the G allele tags a subgroup of patients with schizophrenia (with enough power so that meta-analysis is positive in schizophrenia) which may have greater chance of improvement by different types of antipsychotics.

How could the G allele of the 5-HT_2A _gene be related to global treatment refractoriness in schizophrenia? The T allele of T102C polymorphism was found associated with a higher expression of 5-HT_2A _mRNA and protein [32,33]. Because the T allele theoretically corresponds to the A allele of the -1438A/G polymorphism [34], patients with the G allele might have lower brain densities of 5-HT_2A _receptors. Extensive convergent studies showed that 5-HT_2A _receptor blockade is useful in the treatment of psychosis [35]. Whether the G allele-associated 5-HT_2A _down expression contributes to make schizophrenic patients more prone to respond to antipsychotic treatment is an interesting possibility that deserves further investigations. The gene coding for the D2 dopamine receptor remains a core candidate gene for pharmacogenetic studies devoted to schizophrenia, as all antipsychotics share a variable but significant affinity for this receptor [4].

## Conclusion

Using a TDT approach, the G allele of the -1438A/G polymorphism of the gene coding for the 5-HT_2A _receptor was found to be transmitted in excess in a subgroup of schizophrenic patients who were good responders to first generation antipsychotics (mainly haloperidol), the specificity of this finding (transmission of the vulnerability allele from parents to the proband, rather than direct association) being counterbalanced by the limited number of informative patients.

## Competing interests

This research was supported by grants from INSERM/DPGRF. The first investigator received financial support from the psychiatric hospital of Cheraga (Algeria).

## Authors' contributions

DB recruited the sample, made the interview and participated in the genetic analyses, NH supervised the clinical assessments and proposed a first draft of the manuscript, CB made the genetic analyses, NR supervised the statistical analyses of the genetic approach, MH supervised the manuscript and participated in funds raising, FK organised the recruitment in his department, PG made the design of the study, corrected the manuscript, and organised the submission of this manuscript. All authors read and approved the final manuscript.

## Pre-publication history

The pre-publication history for this paper can be accessed here:


